# Plasmacytoid dendritic cells contribute to vascular endothelial dysfunction in type 2 diabetes

**DOI:** 10.3389/fcvm.2023.1222243

**Published:** 2023-11-29

**Authors:** K. Alluri, B. Srinivas, S. Belmadani, K. Matrougui

**Affiliations:** Department of Physiological Sciences, EVMS, Norfolk, VA, United States

**Keywords:** type 2 diabetes, Plasmacytoid dendritic cells (pDCs), endothelial dysfunction, inflammation, signaling

## Abstract

**Objective:**

Type 2 diabetes (T2D) is associated with an increased risk of cardiovascular disease due to macro- and microvascular dysfunction. This study aimed to investigate the potential involvement of plasmacytoid dendritic cells (pDCs) in T2D-related vascular dysfunction.

**Approach and results:**

pDCs were isolated from db/db and control mice. It was found that pDCs from db/db mice impaired endothelial cell eNOS phosphorylation in response to ATP and decreased vascular endothelium-dependent relaxation compared to pDCs from control mice. Moreover, isolated CD4+ cells from control mice, when stimulated overnight with high glucose and lipids, and isolated pDCs from db/db mice, display elevated levels of ER stress, inflammation, and apoptosis markers. Flow cytometry revealed that pDC frequency was higher in db/db mice than in controls. *In vivo*, the reduction of pDCs using anti-PDCA-1 antibodies in male and female db/db mice for 4 weeks significantly improved vascular endothelial function and eNOS phosphorylation.

**Conclusion:**

pDCs may contribute to vascular dysfunction in T2D by impairing endothelial cell function. Targeting pDCs with anti-PDCA-1 antibodies may represent a promising therapeutic strategy for improving vascular endothelial function in T2D patients. This study provides new insights into the pathogenesis of T2D-related vascular dysfunction and highlights the potential of immunomodulatory therapies for treating this complication. Further studies are warranted to explore the clinical potential of this approach.

## Introduction

Type 2 diabetes is a chronic, complex metabolic disease that involves multiple pathways and mechanisms. It is well-established that type 2 diabetes is associated with vascular endothelial dysfunction (1–9). Endothelial dysfunction is a common complication of type 2 diabetes. It is thought to play a significant role in the increased risk of cardiovascular diseases and other complications associated with type 2 diabetes. It has been reported that insulin resistance, chronic hyperglycemia, oxidative stress, inflammation, advanced glycation end products, dyslipidemia, impaired insulin signaling and nitric oxide production, and alteration in endothelial cell structure contribute to vascular endothelial dysfunction in type 2 diabetes (10). For instance, it has been reported that hyperglycemia impairs vascular endothelial function through protein kinase C beta (11, 12), Mitogen-activated protein kinase (13), reactive oxygen species through disrupting nitric oxide signaling and reducing its production and availability (14), and caveolae (15).

The impact of the immune system on vascular endothelial dysfunction in type 2 diabetes is still unknown. The immune system plays a significant role in the pathogenesis of type 2 diabetes (16, 17). The innate immune system comprises various cells, including macrophages and dendritic cells, that recognize and respond to foreign pathogens and damaged cells. Specifically, the role of the plasmacytoid dendritic cells (pDCs) on vascular dysfunction in type 2 diabetes is unexplored. The pDCs are a subset of immune cells that play a critical role in the body's immune response to viral infections and cancer. In recent years, research has suggested that pDCs may also be involved in developing and progressing chronic inflammatory and cardiovascular diseases such as type 2 diabetes and its complications.

Thus, obese post-menopausal women with type 2 diabetes have higher levels of circulating conventional dendritic cells (cDCs) compared to age-matched healthy women, while a smaller increase was observed for pDCs (18). These findings suggest that DCs may play a role in the development of pathological vascular remodeling in type 2 diabetes (18). In individuals with type 2 diabetes, these cells are activated by obesity and high blood sugar levels, releasing pro-inflammatory cytokines and chemokines (16, 19, 20). The mechanisms by which the immune system contributes to the development and progression of type 2 diabetes are not fully understood. However, it has been reported that chronic low-grade inflammation induced by immune system activation plays a critical role in promoting insulin resistance and beta-cell dysfunction, leading to diabetes and related cardiovascular complications (17, 19, 21, 22). In the present study, we sought to determine the impact of pDCs in type 2 diabetes-induced vascular endothelial dysfunction. Thus, we assessed the frequency and function of pDC and the effects of depleting pDCs on micro- and macrovascular endothelial dysfunction in type 2 diabetic mice.

## Material and methods

All the experimental procedures conformed to the National Institutes of Health Guide for the Care and Use of Laboratory Animals. They were approved by the Institutional Animal Care and Use Committee at the Eastern Virginia Medical School (Norfolk, VA). Eight weeks old type 2 diabetic male and female mice (db/db) and their controls (db/het) were obtained from Jackson Laboratory (Bar Harbor, ME) and were housed in a temperature-controlled room (22 ± 1°C), exposed to 12 h of the dark-light cycle, and fed a standard chow research diet, and deionized water. Mice were euthanized using a 5% isoflurane overdose, followed by heart excision.

### *In-vivo* treatment with anti-mPDCA-1 mAb

Nine to twelve weeks old Male and female mice were randomly divided into 6 groups: Group1: Male db/het mice without treatment (control); Group 2: Male db/db mice without treatment (control); Group 3: Male db/db mice treated with anti-mPDCA-1[130-092-550, miltenyibiotech, Lot No; 5210305844] (dose: 500 μg/mouse, intraperitoneal injection every other day) for 1 month; Group 4: Female db/het mice without treatment (control); Group 5: Female db/db mice without treatment (control); Group 6: Female db/db mice treated with anti-mPDCA-1[130-092-550, Miltenyi Biotech, Lot No; 5210305844] (dose: 500 μg/mouse, intraperitoneal injection every other day) for 1 month.

### Gene expression

Total RNA isolation from db/het, db/db, and anti-mPDCA-1 mAb treated db/db mice using MagMAX RNA isolation kit (Ref: AM1830, Lot 01257092, Thermo Fisher Scientific). The cDNA was synthesized using the High-Capacity cDNA Reverse Transcription kit (Iscript Bio-Rad # 1708840), following the manufacturer’s instructions. Quantitative real-time PCR (qRT-PCR) was carried out using TaqMan Fast Advanced Master mix (Cat# 4444556 Thermo scientific) with specific probes for the amplification for ATF6 (Mm01295319_m1#PN2034377), PERK (Eit2aK3, Mm00438700_m1#PN435137), CHOP (Ddit307295308_m1#PN4351370), IRE1(Ern1, Mm00470233_m1#PN4351370), XBP1(Mm00457357_m1#PN2040082), COX2(Mm03294838_g1#4331182), iNOS(Mm00440502_m1#PN4351370), Caspase3(Mm01195085_m1, Lot2025341), Caspase12(Mm00438038_m1, Lot 1966214), Thbs1(Mm00449032_g1 lot P220917), and GAPDH (Mm99999915_g1#PN1996679) was used for normalization. The relative mRNA expression was calculated using the 2-ΔΔ CT method.

### Vascular endothelial function

Mesenteric resistance arteries (MRA) and aorta reactivity in all groups of mice was evaluated as previously reported (7, 23, 24). MRA and aorta from all groups of mice were immediately placed in cold PSS solution, carefully cleared of perivascular fat and connective tissue, and cut into rings (2 mm in length). The arteries were mounted in a small vessel-chamber myograph (DMT myograph; ADInstruments Ltd., Oxford, U.K.) to measure isometric tension. After 30 min equilibration period in PSS solution bubbled with CO2 at 37°C (pH = 7.4), arteries were stretched to their optimal lumen diameter for active tension development. After 1-h incubation, arteries rings were pre-constricted with phenylephrine (PE, 10^−8^–10^−4^ M), and then when a steady maximal contraction was reached, cumulative dose-response curves were obtained for acetylcholine (ACh, 10^−8 ^–10^−4^ M) and sodium nitroprusside (SNP, 10^−8^–10^−4^ M).

### Flow cytometry

Spleens were collected aseptically, and splenocytes were prepared by mechanical disruption of spleens from db/het, db/db, and db/db treated with anti-mPDCA-1mice. Briefly, the spleens were cut into small pieces and were strained using a 70-μm cell strainer. Spleen slices were transferred to a petri dish and incubated with collagenase D (cat # 11088858001 and DNase-I (cat 10104159001) from Roche Diagnostics, USA, for 20 min in 1% (FBS) complete media (RPMI) at 37°C on a shaker. After incubation, splenic contents were collected and washed with MACS buffer (Miltenyi Biotec, Lot No # 7211000389) at 2,000 rpm at 4°C for 5 min. Spleen fragments were gently pressed with a syringe plunger to remove clumps for clear separation and passed through a 70 μM filter in MACS buffer.

Cells were centrifuged at 2,000 rpm, and RBC in the pellet was cleared in lysis buffer (8.3 g/L ammonium chloride in 0.01M Tris-HCl buffer on ice for 5 min incubation. Cells were washed with MACS buffer at 2,000 rpm for 5 min at 4°C. The cell pellet was resuspended in 2 ml MACS buffer, and cell count was done in a cello meter using AOPI dye. 1× 106 cells were stained with fluorochrome anti-markers to quantify dendritic cells and T-reg cells on ice for 30 min per the manufacturer's recommendation. After incubation, cells were washed with MACS buffer at 2,000 rpm for 5 min at 4°C. The supernatant was decanted, and the pellet was resuspended in 200 μl MACS buffer. Stained Single-cell suspensions were acquired in Cytekaurora DXP8color, and unmixing and data were analyzed using Cytek software. The following antibodies were obtained from Biolegend: anti-CD45 (clone 30-F11, cat # 103135), anti-F4/80 (clone BM8, cat # 123116), anti-CD3 (clone 17A2, cat # 100268), anti-CD4 (clone KG 1.5, cat # 100473), anti-CD25 (clone 3C7, cat # 101904), anti-CD11c (clone N418 cat # 117363), anti-CD11b(M1/70), anti-MHC-II (M5/114.15.2, cat # 107647), anti-CD8a (clone 53-6.7, cat # 100725), anti-CD103 (clone 2E7, cat # 121425), anti-CD45R (clone RA3-6B2, cat# 103261),True Stain Monocyte Blocker (Cat # 426103), Zombie NIR (cat # 423106) for live and dead separation and anti-Siglec-H (clone 440C, cat # 566581), Brilliant Stain Buffer Plus (Cat # 566385) was purchased from BD Bioscience.

### Exercise exhaustion test

After 3 days of acclimatization to treadmill exercise, an exhaustion test was performed in the experimental groups of mice. Mice ran (flat 180°) on the treadmill (Ugo Basile SRL, Model 47300-001, Italy), starting at a warm-up speed of 5 m/min for 4 min, after which the rate was increased to 14 m/min for 2 min. Every next 2 min, the speed was gradually increased by 2 m/min until the mouse was exhausted. Exhaustion was defined as the inability of the mouse to return to running within 10 s of direct contact with an electric stimulus grid, and running distance was calculated.

### Body weight

We measured body weight in all mice groups at the end of the 4th week.

### Nitric oxide synthase

• The P-eNOS activity was measured in the MRA lysates from all groups of mice and lysates of endothelial cells using the mouse P-eNOS enzyme-linked Immunosorbent assay (ELISA) kit according to the manufacturer's protocol (ab279779, Abcam, Waltham, MA. USA). Optical density at 450 nm was used to calculate the P-eNOS activity in the samples, and results were expressed P-eNOS/Total eNOS in (A.U).
• Western blot analysis: As previously reported, phosphorylated and total eNOS and beta-actin expression were determined in the MRA lysates from all mice groups and endothelial cell lysates using Western Blot analysis (7).

### Immune CD4+ isolation

CD4 cells are isolated from db/het and db/db mice using a CD4 T cell isolation kit from Biolegend as per manufacturer protocol (cat # 480006, San Diego, CA, United States) and cultured in RPMI 1640 with 1% penicillin-streptomycin. 1% Glutamax and 10% FBS media. Cells (400,000–500,000) were stimulated with high glucose (25 mM) 2% of lipids mixture overnight, followed by RNA isolation for gene expression studies.

### *In vitro* data “incubation of culture endothelial cells and arteries with pDCs.”

#### Endothelial cells

Human Cardiac Microvascular Endothelial Cells (HCMEC, Lonza, US) were used in our experiments. Cells were cultured in a cell culture incubator in EGMTM -2 MV Microvascular Endothelial Cell Growth Medium-2 Bullet Kit (Lonza, US) at 37°C. HCMEC used for the experiments were from passages 3 to 10. Plasmacytoid dendritic cells were isolated using a plasmacytoid dendritic isolation kit from stem cell technologies as per manufacturer protocol (cat # 19764 Vancouver, BC, Canada). Spleen was collected aseptically after we euthanized mice. Then we gently squeezed it and filtered it through a 70 μm cell strainer. Samples were prepared at a 1 × 10^8^ cells/ml cell concentration within a 0.5–2 ml volume range. The isolation cocktail was added at 50 μl/ml and mixed thoroughly. The mixture was then incubated on ice for 15 min. Cells were washed with 1 ml of MACS buffer (PBS + 1% BSA) and centrifuged at 300 g for 10 min. The supernatant was then decanted. One milliliter of MACS buffer was added to the cell pellet, and the pellet was resuspended. A 100 μl/ml biotin cocktail was added and mixed thoroughly, then incubated on ice for 10 min. After incubation, 37.5 μl/ml of magnetic particles were added, mixed, and set on ice for 10 min. The MACS buffer was added to make a total volume of 2.5 ml, and the tube was placed into the magnet for 5 min. We picked up the entire magnet at a stretch and inverted the tube to transfer the enriched cell suspension into a new tube. To the enriched suspension cells, 7 μl/ml of magnetic particles were added and mixed. The mixture was then incubated for 5 min in a magnet. Afterward, the magnet was poured into the tube to collect the plasmacytoid dendritic cells.

EC cells were serum starved in 1% EGMTM -2 MV media for 4 h, stimulated with and without 30 µM of ATP, and seeded with approximately 100,000–200,000 pDCs of db/het and db/db mice for 4 h.

#### pDCs and mesenteric resistance arteries function

Mesenteric resistance arteries from control C57/Bl6 mice were isolated, mounted in a myograph, and exposed to phenylephrine, acetylcholine, and a nitric oxide donor with and without isolated pDCs from db/db mice. The arteries were incubated with the pDCs for 60 min.

### Statistical analysis

Data are expressed as mean ± SEM. Statistical calculations for significant differences were performed using a *t*-test, one-way ANOVA, and *post hoc*. Comparisons are considered significant when *p* < 0.05.

## Results

### Effect of pDCs on eNOS phosphorylation and endothelium-dependent relaxation

**Figure 1:**
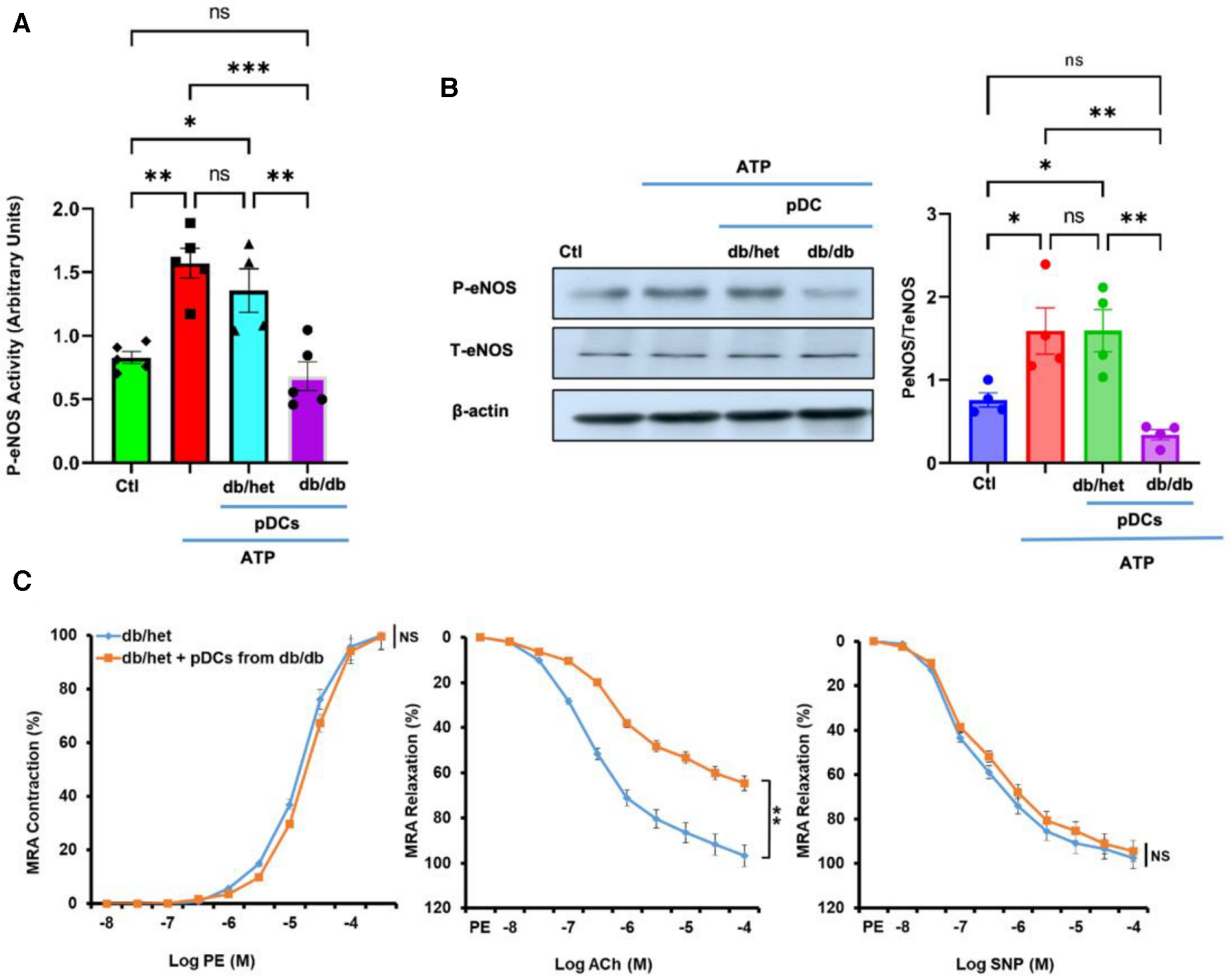
pDCs and eNOS. The pDCs from db/db mice incubated with endothelial cells blunted eNOS phosphorylation in response to ATP compared to pDCs from db/het using kit assay (**A**) and Western blot analysis with cumulative data (**B**). Isolated MRA from db/het reactivity with and without pDCs from db/db mice. pDCs from db/db mice impair MRA endothelium-dependent relaxation but not contractility and endothelium-independent relaxation (**C**) *P < 0.05, **P < 0.01, ***P < 0.001, and ns, not significant, (*n* = 5); one-way ANOVA with Tukey's multiple comparisons for eNOS expression and unpaired *t*-test for MRA endothelium-dependent contraction and relaxation.

Culture microvascular endothelial cells were stimulated with ATP with and without pDCs isolated from db/het and db/db. Using kit assay, our data illustrate that pDCs from db/db mice blunted eNOS phosphorylation in response to ATP compared to pDCs from db/het (Figure 1A). The results were confirmed using Western blot analysis (Figure 1B), indicating that pDCs in db/db contribute to vascular endothelial dysfunction through the eNOS mechanism. These data are also supported by incubating pDCs isolated from db/db mice with mesenteric resistance arteries (MRA) isolated from control mice. Data show that pDCs from db/db mice induce endothelial dysfunction, as indicated by the reduction in endothelium-dependent relaxation (Figure 1C), but no effect on contractility and endothelium-independent relaxation. These *in vitro* studies show that pDCs in db/db are dysfunctional and contribute to endothelial dysfunction.

### ER stress and inflammation in pDCs and CD4+

**Figure 2:**
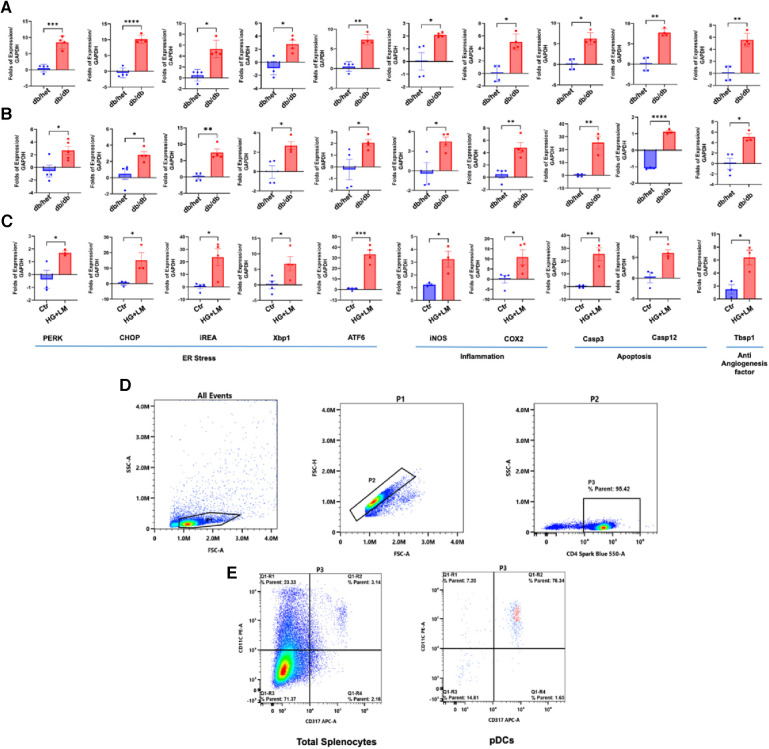
ER stress, inflammation, and anti-angiogenic factors in pDCs and CD4+ populations. The qPCR for ER stress (PERK, CHOP, IREa1, Xbp1s, and ATF6), inflammation (iNOS and Cox2), and anti-angiogenic factors (thrombospondin-1 “Tbsp1”) in CD4+ (**A**) and (**B**) pDCs (subpopulation) isolated from db/het and db/db mice. (**C**) CD4+ cells were isolated from control male mice and stimulated overnight with high glucose (25 mM) and 2% lipids mixture. (**D**,**E**) Representative flow cytometry charts for CD4+ and pDCs purity. *P < 0.05, **P < 0.01, ***P < 0.001, and ****P < 0.0001, (*n* = 3–5); unpaired *t* test for dbhet and db/db; paired *t* test for CD4+ cells treated with high glucose and lipids mixture.

To determine whether only pDCs from db/db are dysfunctional, we isolated the CD4+ population and pDCs from male db/db mice and their controls. These CD4+ and pDCs were subjected to qPCR for ER stress, inflammation, and anti-angiogenic genes (Figure 2). Data show ER stress, inflammation, and anti-angiogenesis markers induction in CD4+ and pDCs isolated from db/db compared to their controls (Figures 2A,B). The data presented above provide further evidence supporting the findings that both pDCs and CD4+ populations are dysfunctional in type 2 diabetes. This suggests that the immune system is compromised in type 2 diabetes. We demonstrated the dysfunctionality of CD4+ and pDC populations in type 2 diabetes. However, due to the low number of pDC and the challenges of culturing them overnight, especially with a high glucose and lipid mixture, we isolated CD4+ cells from male control mice (db/het) and cultured them. Subsequently, we stimulated the CD4+ overnight with a high glucose and lipid mixture to replicate the conditions of type 2 diabetes. The data showed high glucose and lipids mixture induced ER stress (PERK, CHOP, IREA, Xbp1, and ATF6), inflammation (iNOS and Cox2), and anti-angiogenic factors (Tbsp1) (Figure 2C), suggesting that in type 2 diabetes, hyperglycemia and hyperlipidemia contribute to the immune cells' dysregulation. Figure 2D shows the purity of pDCs and CD4+ isolation.

### Effect of reduced pDCs on body weight and exercise

**Figure 3:**
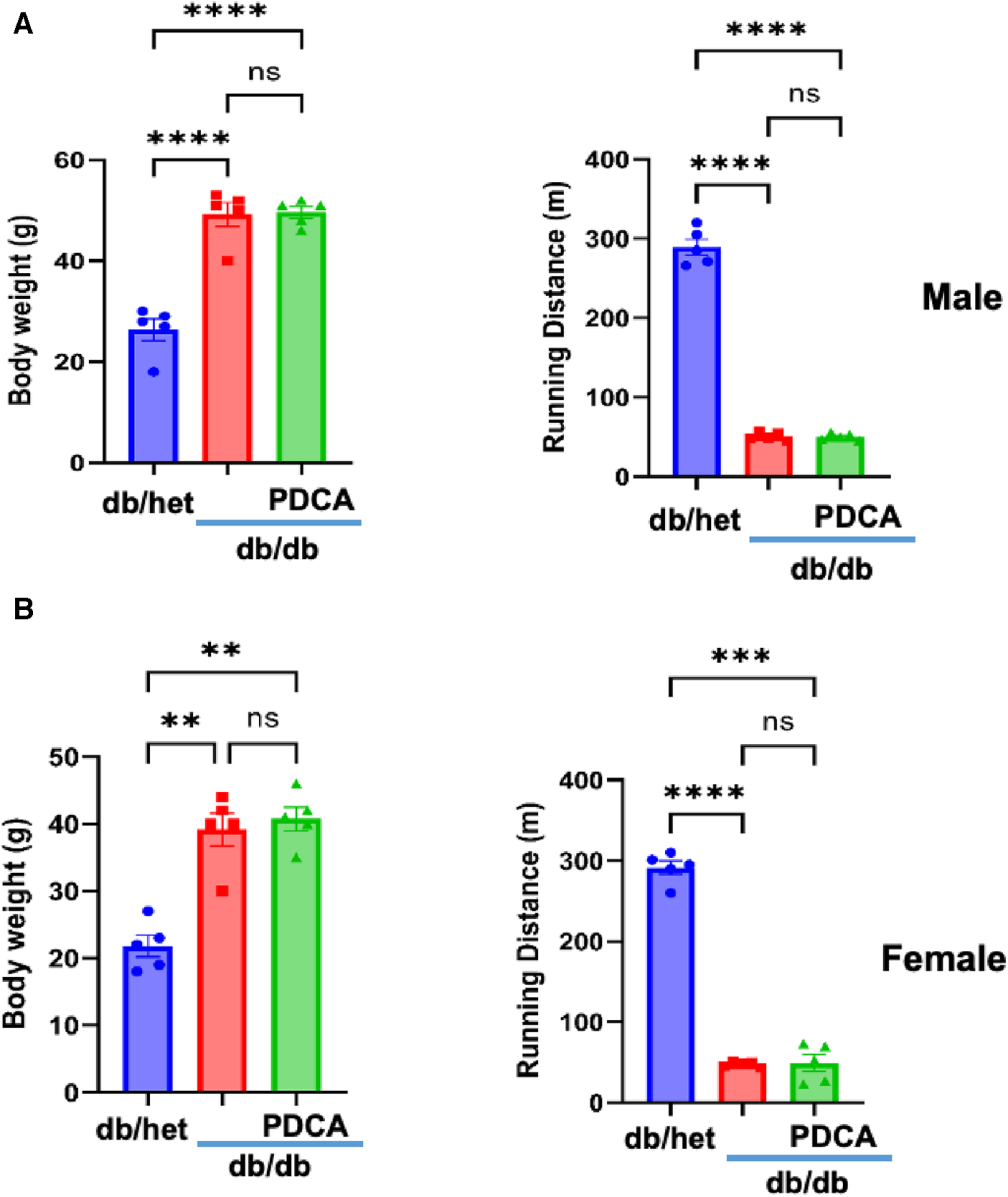
Relationship between body weight and exercise tolerance. Body weight and exercise tolerance (running distance) in male (**A**) and female mice (**B**) db/het, db/db, and db/db mice treated with anti-PDCA to reduce pDCs. **P < 0.01, ***P < 0.001, ****P < 0.0001, and ns, not significant, (*n* = 5); one-way ANOVA with Tukey's multiple comparison.

It is well-established that type 2 diabetes (T2D) is associated with an increased risk of cardiovascular disease due to macro- and microvascular dysfunction. After determining that pDCs in db/db mice are dysfunctional and impair eNOS phosphorylation *in vitro*, we depleted pDCs *in vivo* in db/db mice for 4 weeks. Male and female mice's body weight and running distance did not change between db/db and anti-PDCA-treated db/db mice. However, a significant difference was observed with db/het mice (Figures 3A,B).

### Dendritic cells frequency

**Figure 4:**
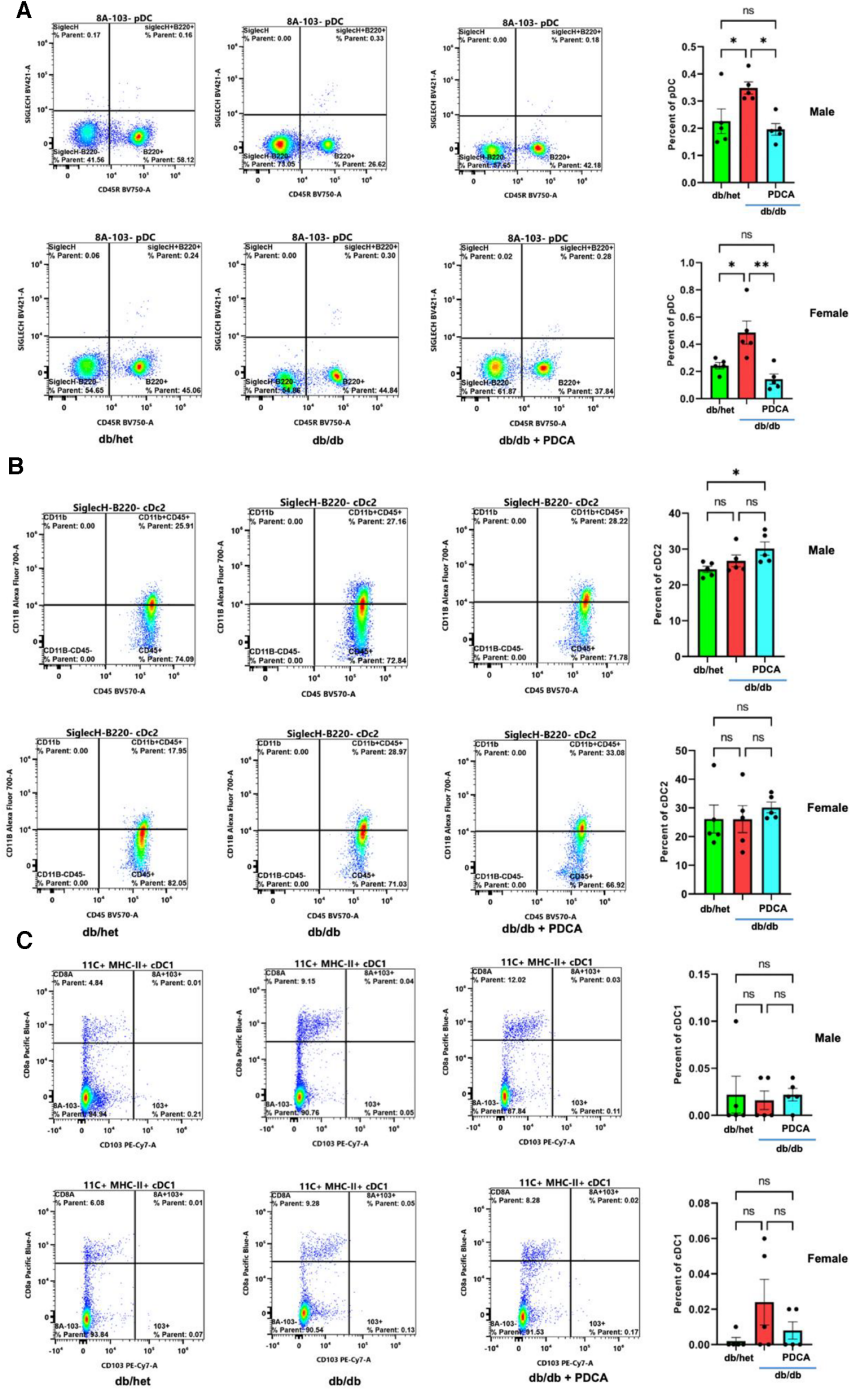
Flow cytometry for dendritic cells. Representative flow cytometry charts and cumulative data for pDCs (**A**), cDC2 (**B**), and cDC1 (**C**) in spleen isolated from db/het, db/db, and db/db treated with anti-PDCA. *P < 0.05, **P < 0.01, and ns, not significant, (*n* = 5); one-way ANOVA with Tukey's multiple comparison.

The immune system plays a significant role in diabetes and cardiovascular complications. However, the profile/frequency and the role of plasmacytoid dendritic cells on diabetes-induced vascular complications have not yet been investigated. Using flow cytometry, we found the number of plasmacytoid dendritic cells “pDCs” was significantly increased in db/db male and female mice compared to their controls and male and female db/db mice treated with anti-PDCA mAb (Figure 4A). The number of myeloid/conventional DC1 “cDC1” and myeloid/conventional DC2 “cDC2” was similar between all male and female groups of mice (Figures 4B,C). The data illustrate that type 2 diabetes increases the number of pDCs in both male and female mice but not cDC1 and cDC2 (Figure 4).

### Effect of *in vivo* pDCs reduction on vascular endothelial function in type 2 diabetes

**Figure 5:**
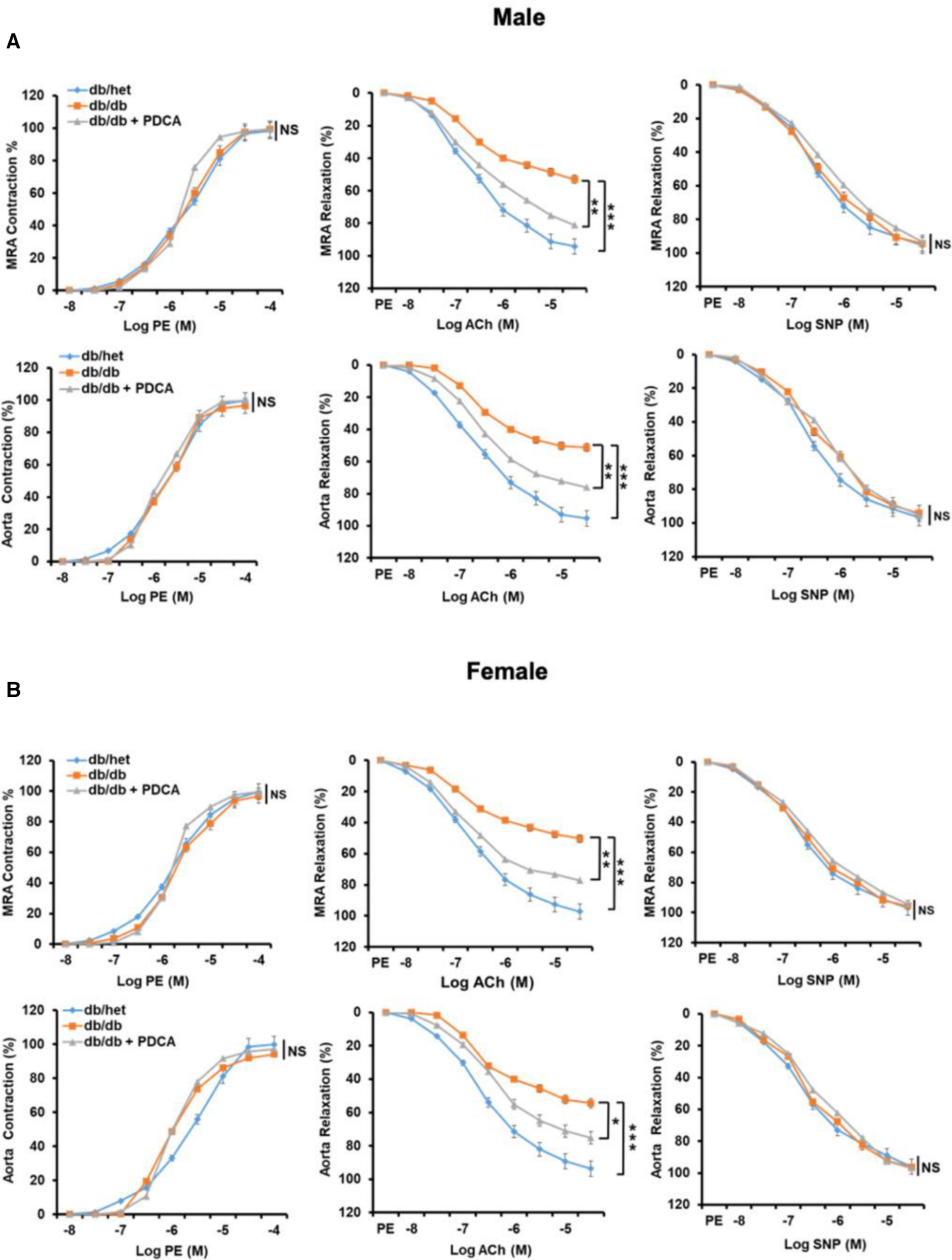
Mesenteric resistance arteries (MRA) and aorta reactivity. Microvascular (MRA) and macrovascular (aorta) contractility in response to sympathetic stimulation (Phenylephrine, PE), endothelium-dependent and independent relaxation in response to acetylcholine (ACh) and sodium nitroprusside (SNP), respectively, in male (A) and female (B) db/het, db/db, and db/db mice treated with anti-PDCA-1 for 1 month. *P < 0.05, **P < 0.01 and ***P < 0.001, and ns, not significant, (*n* = 5); one-way ANOVA with Tukey's *post hoc* multiple comparison.st hoc multiple comparison.

It is well-established that type 2 diabetes is associated with vascular endothelial dysfunction. The contractility and relaxation-endothelium independent in response to phenylephrine and nitric oxide donor in mesenteric resistance artery (MRA) and aorta were similar in all groups of mice (Figure 5). The endothelium-dependent relaxation of MRA and aorta from male and female db/db mice were significantly reduced (Figure 5). Interestingly, the reduction of pDCs with anti-PDCA-1 treatment greatly improved MRA and aorta endothelium-dependent relaxation in both male and female db/db mice (Figure 5). These results suggest that the pDCs significantly contribute to micro- and macrovascular endothelial dysfunction in type 2 diabetes.

### pDCs' role in vascular endothelial dysfunction in type 2 diabetes

**Figure 6:**
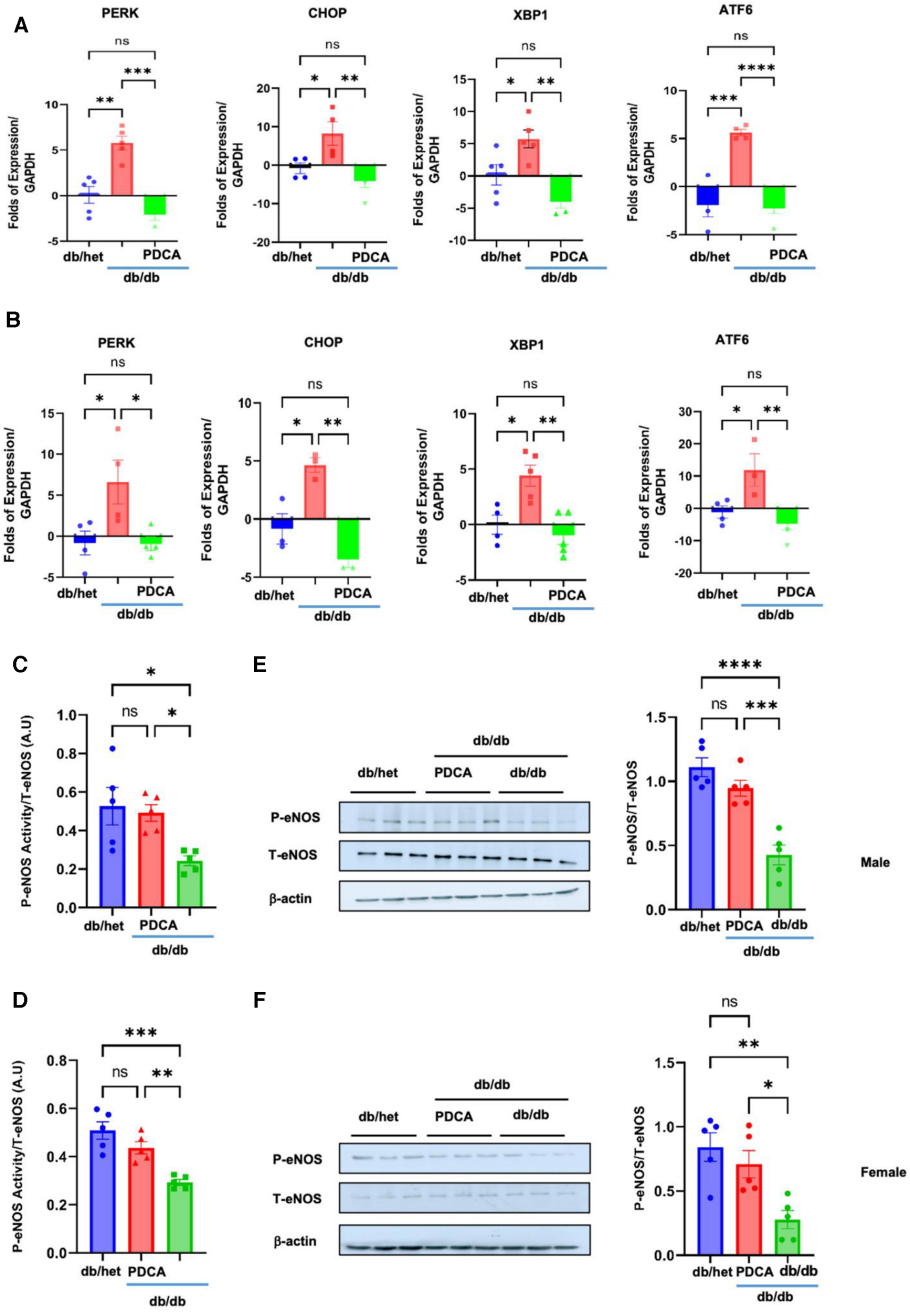
ER stress and eNOS phosphorylation in MRA. The ER stress (PERK, CHOP, Xbp1, and ATF6) induction in MRA from male (**A**) and female mice (**B**) Reduction in eNOS phosphorylation in the MRA from male and female db/db compared to db/het and db/db mice treated with anti-PDCA mAb using kit assay (**C**,**D**) and Western blot analysis with cumulative data (**E**,**F**). *P < 0.05, **P < 0.01, ***P < 0.001, ****P < 0.0001, and ns, not significant, (*n* = 5); one-way ANOVA with Tukey's multiple comparison.

To determine how pDCs contribute to vascular endothelial dysfunction, we assessed the expression of the ER stress markers and eNOS phosphorylation level in the MRA. Our data revealed the induction of ER stress and reduction in eNOS phosphorylation (using kit assay and Western blot analysis) in the MRA from db/db compared to db/het and db/db treated with anti-PDCA (Figure 6), suggesting that pDCs contribute to vascular endothelial dysfunction likely through the induction of the ER stress and reduction in eNOS phosphorylation. Altogether, our *in vitro* and *in vivo* data support that pDCs are dysfunctional in type 2 diabetes and contribute to vascular endothelial dysfunction and potentially cardiovascular diseases related to diabetes.

## Discussion

The present study aimed to determine whether plasmacytoid dendritic cells (pDCs) contribute to vascular endothelial dysfunction in male and female type 2 diabetic mice. Our findings indicate that the frequency and function of pDCs are impaired in type 2 diabetes due to inflammation and endoplasmic reticulum stress induction. We also found that in type 2 diabetes, pDCs cause vascular endothelial dysfunction by inhibiting nitric oxide synthase (eNOS)-dependent mechanism. Notably, reducing pDCs in type 2 diabetes significantly improved vascular endothelial function. These results suggest that targeting the pDC subpopulation could be a promising therapeutic approach for reversing diabetes-induced vascular dysfunction.

The endothelium is a thin layer of cells that lines the inner surface of blood vessels, and it plays a crucial role in regulating vascular function. Type 2 diabetes is a significant and complex health issue. One of its common complications is vascular endothelial dysfunction, which is a critical factor in developing various complications associated with the condition. In type 2 diabetes, the endothelium undergoes structural and functional changes that impair its ability to regulate vascular tone, blood flow, and coagulation. As a result, it plays an essential role in the increased risk of cardiovascular disease and other complications associated with type 2 diabetes. Multiple studies have reported several mechanisms contributing to vascular endothelial dysfunction in type 2 diabetes (1–9). These mechanisms include oxidative stress, endoplasmic reticulum stress, inflammation, tyrosine kinases, advanced glycation end products, poly(ADP-ribose) polymerase 1, impaired nitric oxide (NO) production, insulin signaling, hyperglycemia, and hyperlipidemia (7, 9, 25–29).

While type 2 diabetes was traditionally thought to be solely a disorder of glucose and lipid metabolism, emerging evidence suggests that the immune system plays an essential role in the development and progression of this condition (16, 17). It has been reported that the immune cells of people with type 2 diabetes, such as *T* cells, B cells, and macrophages, are dysfunctional and contribute to the chronic inflammation seen in type 2 diabetes (16, 17, 30–33).

Dendritic cells ([Plasmacytoid dendritic cells (pDCs), conventional type 1 dendritic cell (cDC1), conventional type 1 dendritic cell (cDC2)] subpopulations) are a type of immune cell that plays a critical role in the initiation and regulation of immune responses. There is emerging evidence that dendritic cells could also contribute to the development and progression of diabetes (34, 35). In type 2 diabetic patients, dendritic cells have been shown to accumulate in adipose tissue and promote the production of pro-inflammatory cytokines, such as IL-6 and TNF-α, by *T* cells (36, 37). This can lead to chronic inflammation, which is thought to contribute to the development of insulin resistance and the progression of type 2 diabetes. However, whether pDCs play an essential role in vascular endothelial dysfunction in type 2 diabetes is still unknown. The present study showed that pDCs frequency and function are compromised in type 2 diabetic male and female mice. The frequency of cDC1 and cDC2 was unaffected in male or female type 2 diabetic mice. Our data show that reducing pDCs in male and female mice with type 2 diabetes did not impact body weight or exercise tolerance for 1 month. The dysfunction of pDCs is related to the induction of inflammation, endoplasmic reticulum stress, apoptosis, and anti-angiogenesis. These data are supported by previous studies reporting the dysfunction of dendritic cells in type 2 diabetes (38, 39).

Our data showed that reducing pDCs significantly improved vascular endothelial function in male and female type 2 diabetic mice. More importantly, pDCs in type 2 diabetic mice contribute to vascular endothelial dysfunction through an eNOS phosphorylation inhibition-dependent mechanism. This concept is supported by *in vitro* data showing that isolated pDCs from db/db mice significantly blunted eNOS phosphorylation in response to ATP compared to isolated pDCs from control mice. While acknowledging that there could be differences between the plasmacytoid dendritic cells (pDCs) of humans and mice, it is well established that these cells release similar cytokines in both species. Our study observed that pDCs from db/db mice affected the phosphorylation of endothelial nitric oxide synthase (eNOS) in human endothelial cells. This finding is consistent with previous publications that have demonstrated histamine exocytosis and the generation and release of arachidonic acid metabolites in response to activation with anti-human IgE in both freshly isolated mouse 3T3 fibroblasts and co-cultured human mast cells with mouse 3T3 fibroblast (40).

Previous data showed that inhibiting pro-inflammatory pathways in dendritic cells and adipose tissue macrophages reverses insulin resistance (41). This supports the concept that dendritic cells are dysfunctional through the induction and release of inflammatory factors.

Furthermore, it has been observed that plasmacytoid dendritic cells (pDCs) from patients with systemic sclerosis produce elevated levels of proinflammatory chemokine CXCL4, which has been linked to the risk and progression of the disease (42). Recent research has established a connection between the abnormal production of CXCL4 and increased expression of interferon gamma-I in pDCs in patients with systemic sclerosis. This study also demonstrated that transient reduction of pDCs can prevent and even reverse skin fibrosis in a chemically induced animal model (43). These findings underscore the need for further investigation into the impact of pDCs on various diseases. The mechanisms by which dysfunctional pDCs contribute to vascular endothelial dysfunction in type 2 diabetes are currently unknown. However, it has been observed that pDCs play a role in the inflammatory environment associated with diabetes. The pDCs release various pro-inflammatory cytokines and chemokines, including interferon-alpha, tumor necrosis factor-alpha, interleukin-6, and other factors.

Furthermore, it should be noted that endothelial microparticles (EMP), released by endothelial cells, can modulate immune cells. Specifically, plasmacytoid dendritic cells (pDC) internalize these EMPs, resulting in a proinflammatory response characterized by the secretion of interleukin-6 (IL-6) and interleukin-8 (IL-8). However, it has been observed that EMPs do not have any impact on conventional dendritic cells (cDCs) (44).

Moreover, the binding process between pDCs and endothelial cells is mediated by αL and α4 integrins on pDCs, as well as intercellular adhesion molecules (ICAM)-1, ICAM-2, and vascular cell adhesion molecule-1 on high endothelial venules (HEVs) (45). Additionally, endothelial cells produce IL-3 and vascular endothelial growth factor (VEGF), which bind to pDCs marker proteins CD123 and BDCA4, promoting pDCs survival (46). Although there is evidence suggesting interactions between pDCs and endothelial cells through ICAM-1/LFA, CD31/CD38, and CD34/CD62l, the chemerin/ChemR23 interaction has been the only one conclusively demonstrated to play a role in the migration process thus far (46). The interaction between endothelial cell-bound chemerin and pDCs ChemR23 appears crucial in facilitating the migration of pDCs from the bloodstream to both lymph nodes and inflamed tissue (47). Moreover, It has been observed that elevated levels of glucose and lipids in the circulation can trigger the production of reactive oxygen species (ROS) or induce a hypoxic state, leading to inflammation in the body. Consequently, this inflammation activates pDCs, causing them to accumulate in adipose tissue and secrete interferon-I (IFN-I) before egressing to lymph nodes (48). These substances can trigger inflammatory responses, ultimately impacting vascular endothelial function in type 2 diabetes. To gain a better understanding, future studies should investigate whether pDCs interact with endothelial cells as previously described (49) and/or release these cytokines and chemokines within the vasculature, both *in vivo* and *in vitro*, and how these releases impair endothelial function, specifically in the context of type 2 diabetes.

Novelty and significance. There is a lack of studies exploring the impact of dendritic cells and the mechanism by which pDCs cause vascular endothelial dysfunction and cardiovascular complications. Although studies are seeking to understand the underlying mechanism of vascular endothelial dysfunction in type 2 diabetes, the proposed research is innovative, in our opinion, because our approach provided direct evidence that pDCs frequency and function are compromised and cause vascular endothelial dysfunction through eNOS and ER stress-dependent mechanism in two different vascular beds.

Emerging evidence from experimental and clinical research indicates that the immune system plays a pivotal role in cardiovascular diseases. Specifically, the significant impact of the subpopulation of dendritic cells (pDCs) on vascular endothelial dysfunction is still unexplored. Lack of such knowledge is a fundamental problem because endothelial dysfunction that causes coronary artery disease will still present a high risk for myocardial infarction in diabetic patients. Thus, our *in vivo* and *in vitro* data indicate a significant role of pDCs in vascular endothelial dysfunction through the eNOS and ER stress-dependent mechanism in two different vascular beds. We don't know how the pDCs in a type 2 diabetic environment impair vascular endothelial function. Further research is needed to investigate whether type 2 diabetes alters the repertoire of adhesion molecules in plasmacytoid dendritic cells (pDCs) and endothelial cells, as well as the effects of these alterations on their interaction and secretion of inflammatory factors.

In terms of novelty and significance, the study provides new insights into the role of pDCs in T2D-related vascular dysfunction, a significant cause of cardiovascular disease in individuals with T2D. The findings suggest that targeting pDCs could be a promising approach for improving vascular endothelial function and preventing or treating T2D complications.

### Limitations

Monoclonal antibodies (mAbs) that target plasmacytoid dendritic cells (pDCs) are a common method to deplete pDCs in animal models and a promising approach in treating various diseases, including autoimmune disorders and cancer. Using mAbs to deplete pDCs in adult type 2 diabetic mice selectively is a significant approach because it can potentially avoid the side effects associated with systemic immunosuppression. The potential non-specific effects of Anti-mPDCA-1 mAb may affect other cell types, leading to unintended consequences. However, using flow cytometry, our data showed a decrease in pDCs but no change in cDC1 and cDC2 frequency, indicating that Anti-mPDCA-1 mAb could be specific to pDCs.

We isolated pDCs from the spleen but not the circulation to study their effect on vascular endothelial function and cultured endothelial cells. Isolating the pDCs from circulation in mice is very challenging. However, pDCs in the spleen can leave and enter the circulation. Dendritic cells are highly mobile cells that can migrate from tissues to draining lymph nodes or other tissues.

## Data Availability

The original data presented in the study are included in the manuscript. Further inquiries can be directed to the corresponding author.
